# Supplementary material for: Near-field detection of gate-tunable anisotropic plasmon polaritons in black phosphorus at terahertz frequencies
Source: Nat Commun. 2024 Mar 15;15:2373. doi: 10.1038/s41467-024-45264-5 (PMC10943022; doi:10.1038/s41467-024-45264-5)
Supplement: Supplementary file 1 — Supplementary Information [file 41467_2024_45264_MOESM1_ESM.pdf]

Supplementary Information

Near-field detection of gate-tunable anisotropic plasmon polaritons in black phosphorus at terahertz frequencies

Eva A. A. Pogna,[1,2] Valentino Pistore,[1] Leonardo Viti,[1] Lianhe Li,[3] Giles A. Davies,[3] Edmund H. Linfield,[3] Miriam S. Vitiello[1]

[1] NEST, CNR - Istituto Nanoscienze and Scuola Normale Superiore, P. San Silvestro 12, 56127 Pisa, Italy
[2] Istituto di Fotonica e Nanotecnologie, Consiglio Nazionale delle Ricerche, Piazza Leonardo da Vinci 32, 20133 Milano, Italy
[3] School of Electronic and Electrical Engineering, University of Leeds, Leeds LS2 9JT, UK

The Supplementary Information consists of 10 sections as follows:

- Supplementary Note 1. Fabrication of hBN-encapsulated black phosphorus photo-detector.

- Supplementary Note 2. Transport characterization of the hBN/bP/hBN field-effect transistors.

- Supplementary Note 3. **Debrides and bP stability: impact of debrides and degradation on bP.**

- Supplementary Note 4. Spatial resolution of the near-field photocurrent nanoscopy.

- Supplementary Note 5. PTE origin of the photovoltage.

- Supplementary Note 6. Modeling of the THz near-field response.

- Supplementary Note 7. Device optimization for photocurrent measurements.

- Supplementary Note 8. Raman spectra.

- Supplementary Note 9. Measurement of the bP polariton wavelength.

- Supplementary Note 10. Numerical simulations of s-SNOM polariton interferometric patterns.

Supplementary Note 1. Fabrication of hBN-encapsulated black phosphorus photodetector

The fabrication steps for preparing the photodetectors for near-field photocurrent measurements are reported in Supplementary Figure 1, together with the optical and SEM images taken during the fabrication of an additional device (device 4). The flakes of hexagonal

boron nitride (hBN) and of black phosphorus (bP) are prepared by mechanical exfoliation and picked up sequentially (top hBN, bP, bottom hBN) with poly-(dimethylsiloxane) (PDMS) and polycarbonate (PC) stamps. The stack is then released at 180 °C on the split gates structure placed on a Si/SiO_2 wafer. This temperature is higher than the glass transition temperature of PC (~150 °C), enabling a better control during transfer thanks to the decreased viscosity of PC. Once the heterostructure is placed on the 30 nm-thick back-gates, the FET contacts are defined by reactive ion etching (RIE, CF_4/O_2 plasma), using a 15 nm aluminum hard mask. Exfoliation may result into bP flakes with edges along the principal crystallographic axis. Therefore, a first selection of the exfoliated flakes, before transfer, can be operated searching for flakes with perpendicular edges and lateral dimension of few microns. To launch and probe the THz polaritons in bP, the top layer hBN must be thin enough[1] to allow detection of the evanescent field from the sample. The source and drain contacts are then realized by electron beam lithography (EBL) followed by metal deposition (Cr/Au, 10/60 nm). Devices 1 and 2 comprise only two gates, identifying a single junction oriented at an angle θ with respect to the AC direction as determined using Raman, see Supplementary Figure 2. The channel length (horizontal extension) of device 1 is L_1= 8.4 ± 0.1 μm, while the average channel width (vertical extension) is W_1= 5.8 ± 0.1 μm (left electrode is 4.7 ± 0.1 μm wide, right electrode is 6.8 ± 0.1 μm wide). The channel length of device 2 is L_2= 5.7 ± 0.1 μm, while the channel width is W_2= 5.9 ± 0.1 μm (left and right electrodes have same size). In both devices, the back gates exceed the dimension of the channel and are separated by a 50 nm gap at the junction. The smaller the gap, the steeper the gradient in the Seebeck coefficient across the gap and larger the PTE photovoltage and its signal-to-noise ratio.

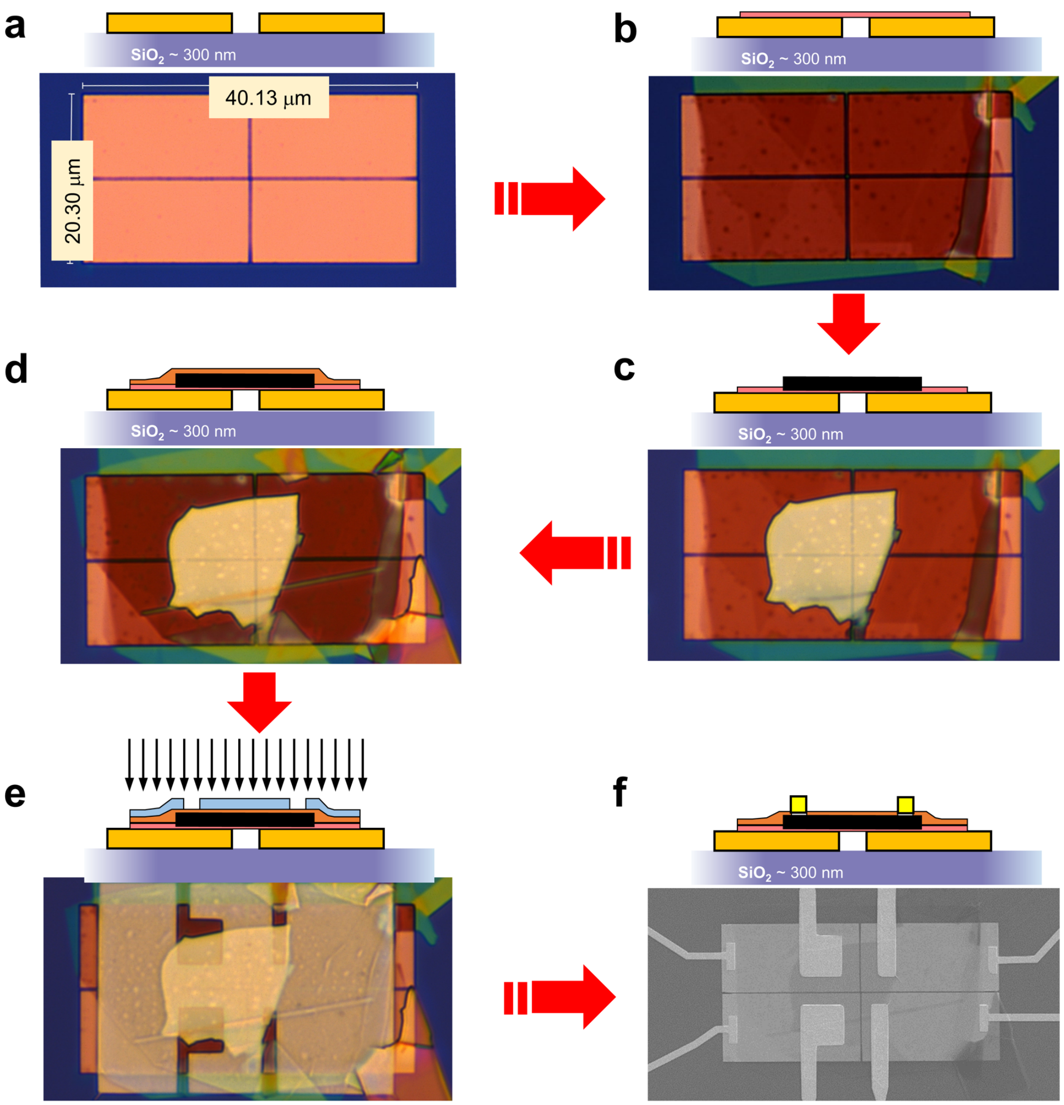

Supplementary Figure 1 | Fabrication steps and images of hBN/bP/hBN FETs for near-field photocurrent measurements. a, Evaporation of the 30 nm thick gold split gates (2 in device 1-2 and 4 in device 3) of the FETs on top of a SiO_2/Si wafer. **b,** Transfer of the exfoliated bottom hBN onto the back-gates. **c,** Transfer of the bP flake on the bottom hBN flake. **d,** Transfer of the top hBN flake onto the bP flake. **e,** Reactive ion etching (RIE, CF_4/O_2 plasma) using a 15 nm thick aluminum mask to remove the top hBN in regions where the FET contacts are placed. **f,** Electron beam lithography followed by metal evaporation of the gates, drain and source electrodes**.** The images of device 4 in panels (a-e) are taken with an optical microscope, while panel (f) reports a SEM image of the final device 4.

Supplementary Note 2. Transport characterization of the hBN/bP/hBN field-effect transistors.

The transport measurements of device 1 and device 2 are reported in Supplementary Figure 3. At zero gate voltages, the channel resistance is R_1= 63 kΩ for the device 1, and R_2=125 kΩ for the device 2.

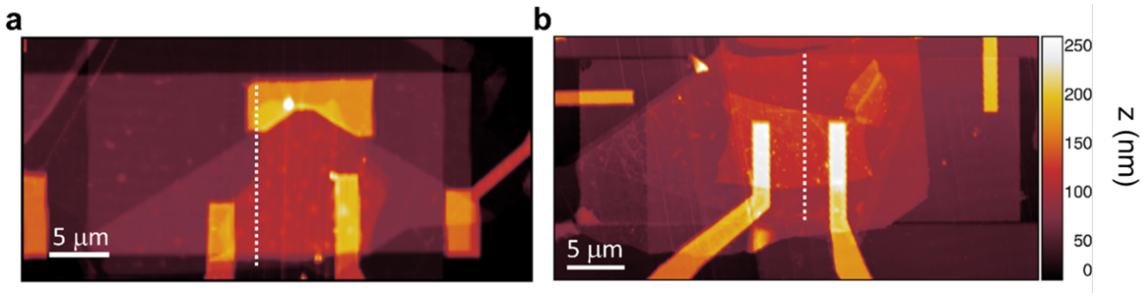

Supplementary Figure 2 | Black phosphorus two-gates photo-transistors. a-b, Atomic force microscope (AFM) image of device 1 (a) and device 2 (b). The junction line is indicated by the white dotted line. The colorbar is expressed in nanometers.

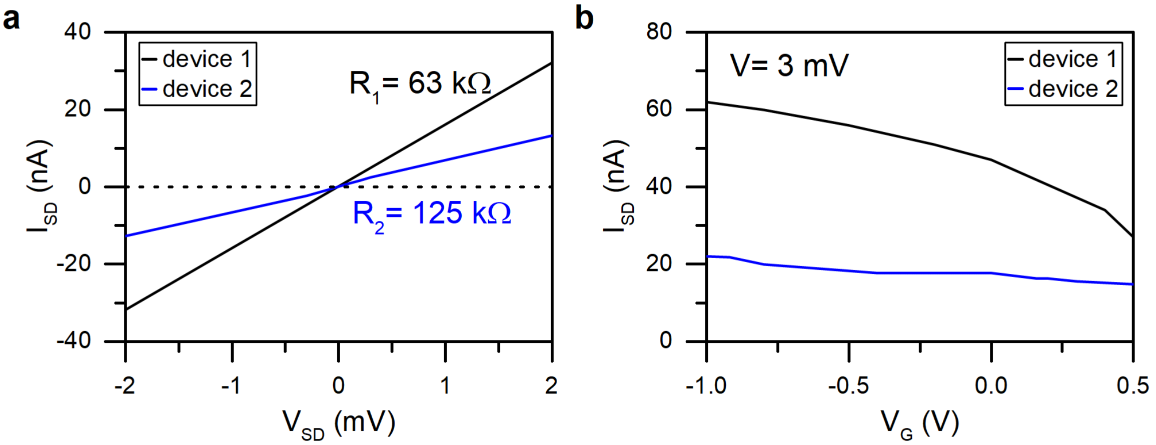

Supplementary Figure 3 | Transport characteristics. a, I-V curves of device 1 (black solid line) and device 2 (blue solid line) collected at zero gate voltages. The channel resistances of device 1 (R_1) and device 2 (R_2) are evaluated as the inverse of the slopes of the related I-V curves. **b,** Room-temperature (RT) transfer characteristics of device 1 (black solid line) and device 2 (blue solid line) measured while sweeping V_G, and while keeping V_{SD} = 3 mV.

The topographic map of device 1, acquired with the SNOM during the photovoltage measurements in Figure 1b, is reported in Supplementary figure 4.

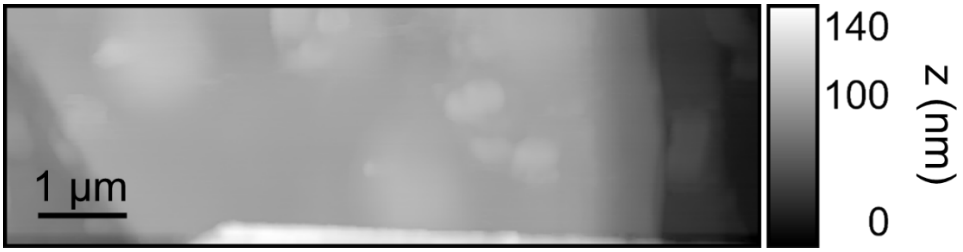

Supplementary Figure 4| AFM topographic map of device 1 corresponding to Figure 1b in nanometers. The scale bar corresponds to 1 µm. The zero height is taken at the substrate surface at the right side of the map.

Device 3 (Supplementary figure 5a) includes four gates, identifying two orthogonal junctions oriented at an angle θ and θ+π/2 with respect to the AC direction. The length of the FET channel in device 3 is L_{ZZ} = 6.6 μm along ZZ direction, and L_{AC} = 4.4 μm along the AC direction. The channels resistance at zero gate voltage are equal to R_{AC} = 44 kΩ along the armchair (AC) direction and R_{ZZ} = 65 kΩ along the zigzag (ZZ) direction (Supplementary figure 5b).

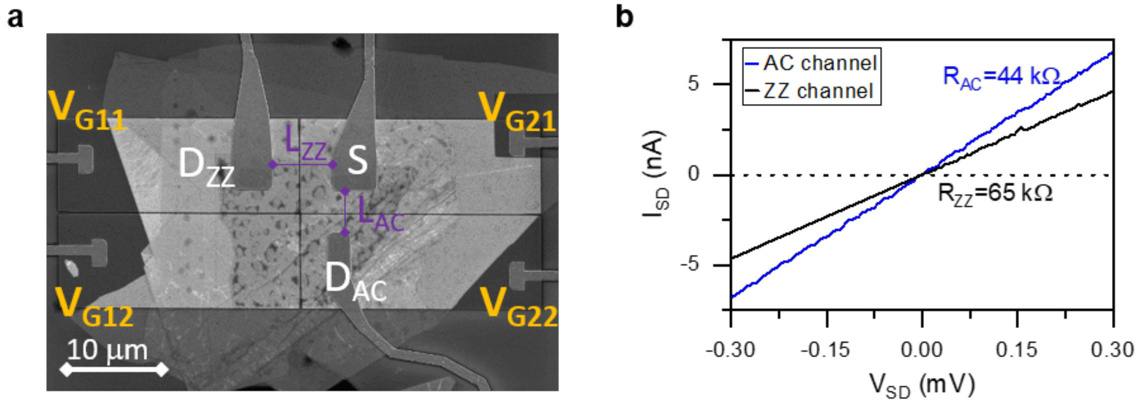

Supplementary figure 5 | Black phosphorus 4-gates photo-transistor. a, Scanning electron microscope (SEM) image of device 3, with four-gates and three readout contacts for measuring the photovoltage detected at the source contact (S) along the armchair (D_{AC}) and zigzag (D_{ZZ}) directions, while modulating the corresponding junctions with the back gates couples (V_{G21}-V_{G22}) and (V_{G11}-V_{G21}) respectively. The channel length along ZZ and AC directions are indicated as L_{ZZ} and L_{AC}. **b,** I-V curve of device 3 along the AC channel (blue line) collected while keeping D_{ZZ} contact floating, and along the ZZ channel (black line) while keeping D_{AC} contact floating. The channel resistances along AC and ZZ directions are evaluated as the inverse of the slope of the I-V curves.

Quantitative determinations of the conductance anisotropy via two-terminal measurements can yield to large errors because of the contribution from contact resistance and device geometry. Accurate determinations of the conductance anisotropy of bP[2,3] give values of about 1.6 ± 0.1, compatible with our observations. A larger conductance anisotropy (up to 7.5) has been reported using edge contacts instead of top contacts[2] and using four-terminal measurements. Supplementary figure 6a shows the transconductance along the ZZ direction, measured while varying the gate voltage V_{G21} (V_{G11}) and while keeping G_{11} (G_{21}) grounded. Supplementary figure 6b plots the transconductance along AC direction, measured by applying a gate voltage V_{G21} (V_{G22}) and while keeping G_{22} (G_{21}) grounded. The largest current modulation is observed along the AC direction applying a bias with gate G_{22}.

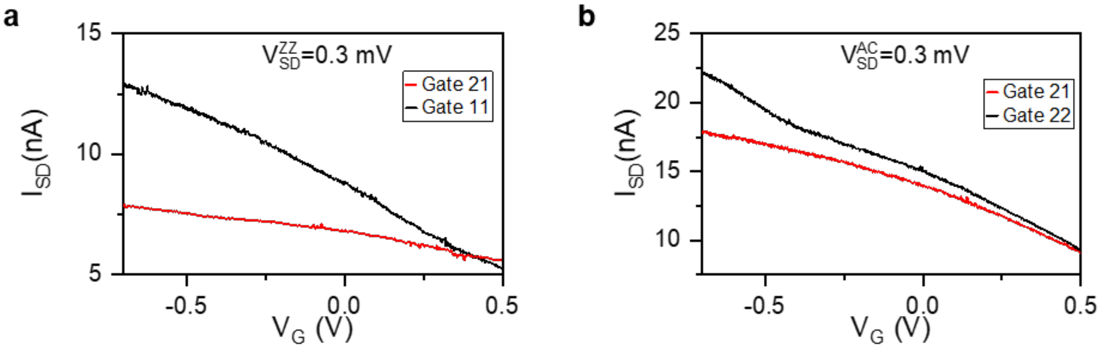

Supplementary figure 6 | Charge carrier density tuning. a, Current along the ZZ channel collected applying a bias V_{ZZ} = 0.3 mV as a function of the gate voltage on gate G_{11} (red solid line) and gate G_{21} (black solid line). **b,** Current along the AC channel collected applying a bias V_{AC} = 0.3 mV as a function of the gate voltage on gate G_{21} (red solid line) and gate G_{22} (black solid line).

The transconductance characteristics of device 1, device 2 (Supplementary figure 3b), and device 3 (Supplementary figure 6), can be used to estimate the carrier density n at zero bias exploiting the relation $n = CV_{th}/(eh)$ where $C= \varepsilon_0\varepsilon_r/d$ is the FET capacitance per unit area, d is the thickness of the bottom hBN layer, V_{th} is the threshold voltage, h is the bP flake thickness and e is the electron charge. The relation $n(V_{th})$ assumes that the charge carrier density is zero at a gate voltage equal to V_{th}. The capacitance per unit area C of the three devices is 2.0×10^{-3} F/m^2 (device 1), 2.4×10^{-3} F/m^2 (device 2), 1.9×10^{-3} F/m^2 (device 3), as estimated using the thickness d extracted by the atomic force microscope (AFM) image and the average permittivity[4] ε_r= 4 for the bottom hBN layer. The threshold voltage V_{th}, retrieved extrapolating the transconductance trends in the linear regime to I_{SD}= 0 nA, is V_{th} = 0.9 V (device 1, Gate 1), V_{th}= 2.4 V (device 2, Gate 1). For device 3, where we measure a finite photovoltage with oscillation along the AC direction, we retrieve distinct values of threshold voltages, depending on the active gate and source-drain channel. Along the ZZ direction, the thresholds are V_{th}= 1.2 V (gate 11), V_{th}= 2.6 V (gate 21). Along the AC direction, the threshold voltages are V_{th}= 1.4 V (gate 21), and V_{th}= 1.3 V (gate 22). The retrieved values of charge carrier density, at zero gate bias, are 6×10^{17} cm^{-3} (device 1), 1×10^{18} cm^{-3} (device 2), 2×10^{18} cm^{-3} (device 3, along ZZ, gate 21), 1×10^{18} cm^{-3} (device 3, along ZZ, gate 11). Since the source-drain current increases by 50% by applying a gate voltage V_{G1}= −2 V in the device 1, and V_{G1}= −3 V in the device 2, we expect carrier densities of ~ 1×10^{18} cm^{-3} (device 1) and ~ 2×10^{18} cm^{-3} (device 2) in the gating conditions

reported in Figure 2. The current between the D_{ZZ} and S contacts of device 3 changes by 10% by sweeping V_{G21} from −0.2 V to −0.6 V and by 41% and 48% by sweeping V_{G11} in the range 0.2 to −0.4 V e 0 to −0.6 V, as shown in Figure 4. The charge carrier density at V_{G11}= −0.2 V and V_{G11}= −0.4 V is estimated as ~1.1 × 10^{18} cm^{-3} and ~1.3 × 10^{18} cm^{-3} from the change in current compared to the V_{G11}= 0 V point.

The PTE photovoltage at the junction can be written as $V_{PTE} = \Delta S_e \Delta T$, where ΔT is the temperature gradient and ΔS_e the Seebeck coefficient difference between the two gated portions of the channel identifying the junction. The Mott equation, describes the dependence of the Seebeck coefficient on the carrier density (on the Fermi energy E_F) that is controlled by the gate bias V_G, as follows:

$$S_e = -\frac{\pi^2 k_B^2 T}{3e}\frac{1}{\sigma}\frac{d\sigma}{d\varepsilon}\bigg|_{\varepsilon=E_F}$$

where k_B is the Boltzmann constant, T is the temperature, e is the electron charge and σ is the channel conductivity. Supplementary figure 7 shows the calculated Seebeck coefficient Se (V_G) extracted from the conductance curve of device 1 and device 2 (Supplementary Fig. 3b), and device 3 (Supplementary Fig. 6a) using the Mott equation and applying the same method described in Ref. [5]

This equation holds in the range in which the mobility is independent from carrier energy.

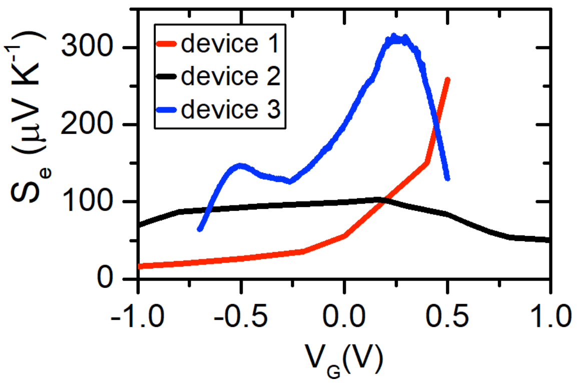

Supplementary figure 7 | Seebeck coefficient as a function of the applied gate. For the device 3 we considered the conductance along the ZZ channel as a function of gate voltage on gate G_{11} from Supplementary Fig. 6a.

Supplementary Note 3. Debrides and bP stability

hBN encapsulation is meant to prevent the degradation of bP flakes by air exposure. In Supplementary figure 8, we compare the AFM images of the encapsulated flake near the 21-11 junction acquired at the beginning and at the end of the photocurrent experiment, after 40 days. The fresh sample shows the presence of debrides trapped during the encapsulation distinguishable by higher (~ 5 nm) height (Supplementary figure 8) and lower (darker) contrast in the near-field images (Supplementary figure 8). Previous investigations have shown that the degradation dynamics of non-encapsulated bP flakes of similar thickness (27 nm) results into formation of nano-sized particles, which appear randomly on the surface, increase in density in few days and then coalesce together, completely altering both the topography and the contrast at mid-infrared frequencies[3]. The near-field microscopy characterization is performed in dark, inert atmosphere purging with N_2 to keep humidity levels < 3%, to further reduce degradation risk.

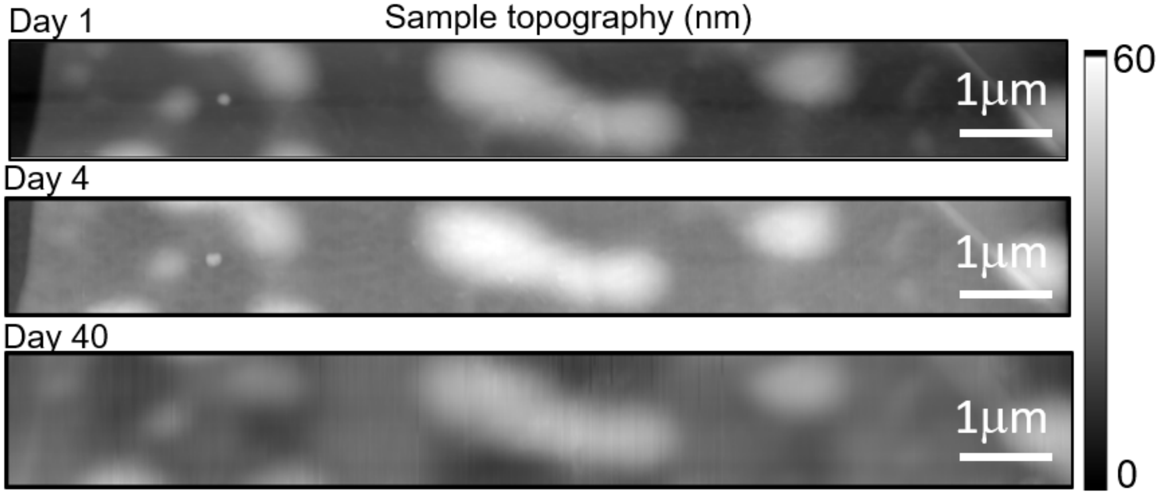

Supplementary figure 8 | Topography evolution during measurements. Time evolution of encapsulated bP topography, around the junction 11-21, during measurements in dark and purged atmosphere from Day 1 (upper panel) to Day 40 (lowest panel).

In our case, the comparison of the topography maps of device 3, as prepared, and after 40 days since the measurements in Supplementary Fig. 8, shows no changes, indicating that the encapsulation is successfully protecting bP from degradation.

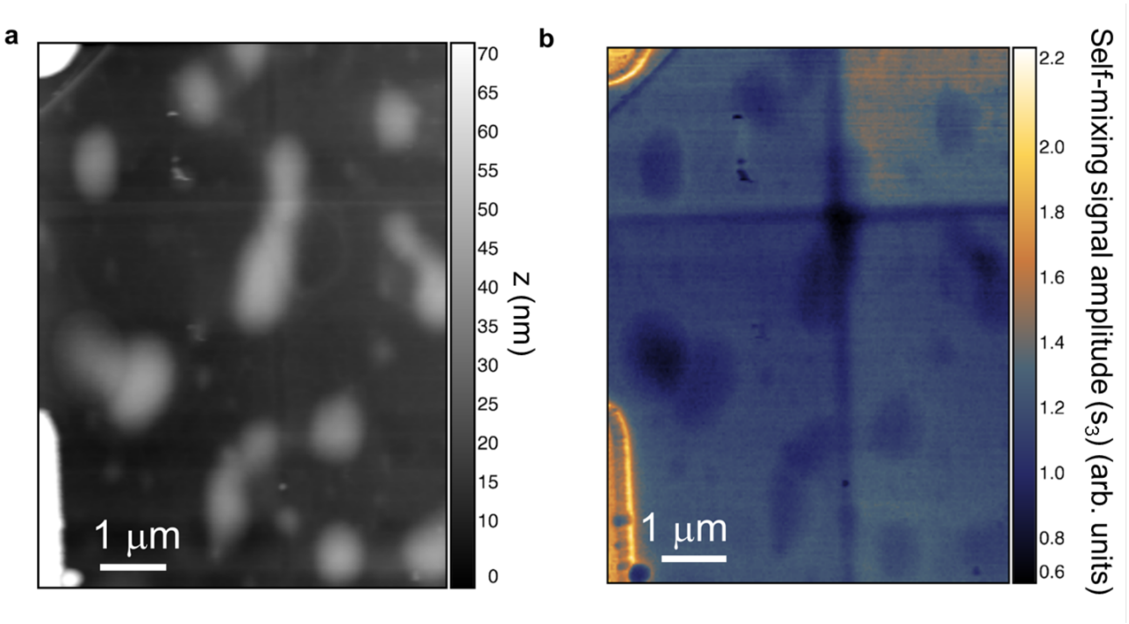

Supplementary figure 9 | Near-field scattering signal and debrides. a, Topographic map of device 3 in nm. **b,** Third harmonics of near-field scattered signal measured by self-mixing interferometry with a 2.01 THz QCL (arbitrary units), showing reduced scattered intensity at the debrides position in the topography. The maps are acquired while applying no bias to the back gates.

The near-field contrast is measured by self-mixing interferometry,[6,7] detecting the voltage of the THz-QCL while performing a near-field map, to self-detect the portion of the light back scattered by the sample-tip system and reinjected into the QCL along the same optical path.[6,7] From the comparison of the topography and self-mixing signal in Supplementary Fig. 9, we see a visible reduction of the THz near-field contrast at the debrides. Correspondingly, at the debrides no near-field PTE photovoltage is detected, likely because the tip is not able to induce the local heating required for producing the PTE current, see Supplementary Fig.10. We consider the presence of finite near-field photovoltage far-away (>1 μm distance) from the junction as a sign of damaging, the presence of a strain pattern is also expected to be associated with a gradient of Seebeck coefficient that can produce a PTE response.

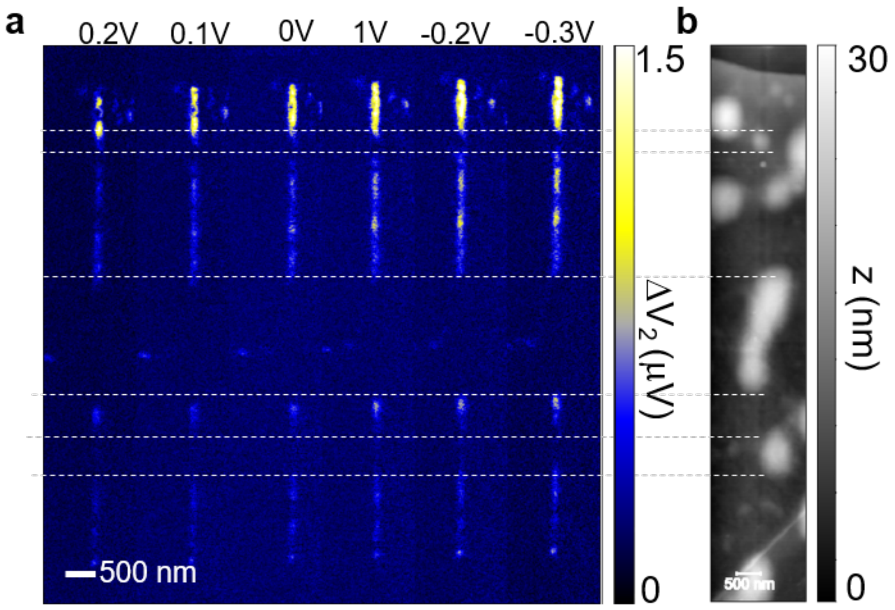

Supplementary figure 10 | Effect of debrides on the photocurrent. a. Second harmonics PTE photovoltage ΔV_2 signal along the AC direction collected at 2.91 THz, with V_{G11} grounded and V_{G21}= {0.2, 0.1, 0, -0.1, -0.2, -0.3} V from left to right. **b.** Topographic map showing the correspondence between the debrides position and the regions where no photovoltage signal is detected.

Few tens of nanometer bP flakes degrade rapidly in air, as investigated also by SNOM[8].

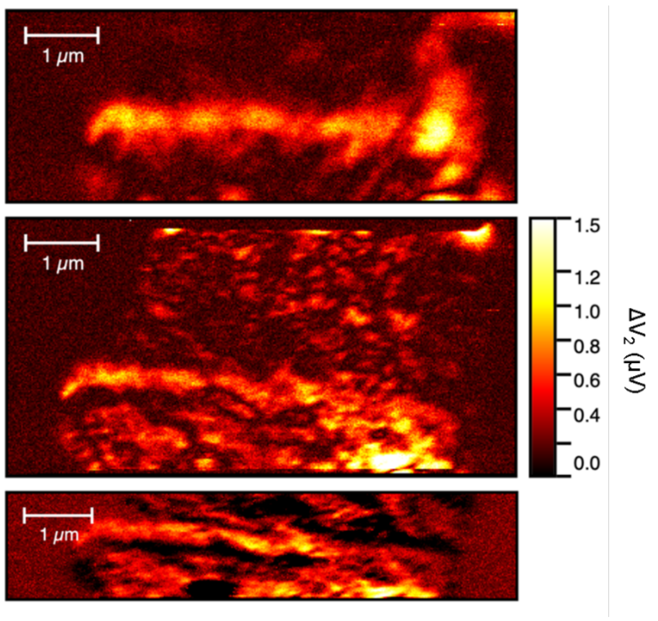

Supplementary figure 11 | Signature of flake degradation in the photovoltage maps measured in three subsequent days (from top to bottom).

Degradation is easily traced in a consistent variation of the topography and optical contrast of nanoflakes. Local strain associated to surface degradation can result in the formation of a disordered PTE current pattern away from the junction, as the one displayed in Supplementary Fig.11. The degradation is clearly observed within few days, with the appearance of PTE signal away from the junction till the signal at the junction completely disappears. In most of the cases, hBN encapsulation has proven successful in preventing degradation induced by air exposure. Therefore, it is important that the flake characterization, including the angle-resolved Raman measurements, is performed after encapsulation.

We have tried to reduce the debris by thermal annealing of the heterostructure, which typically results in a clusterization of small bubbles into larger ones.

Supplementary Note 4. Spatial resolution of the near-field photocurrent nanoscopy

The spatial resolution of the THz near-field photocurrent nanoscopy is estimated by mapping the photovoltage near the drain contact of device 1, on which the photovoltage is expected to jump to zero since the THz field, funneled by the tip, is shielded by the gold electrode (Supplementary Fig.12a).

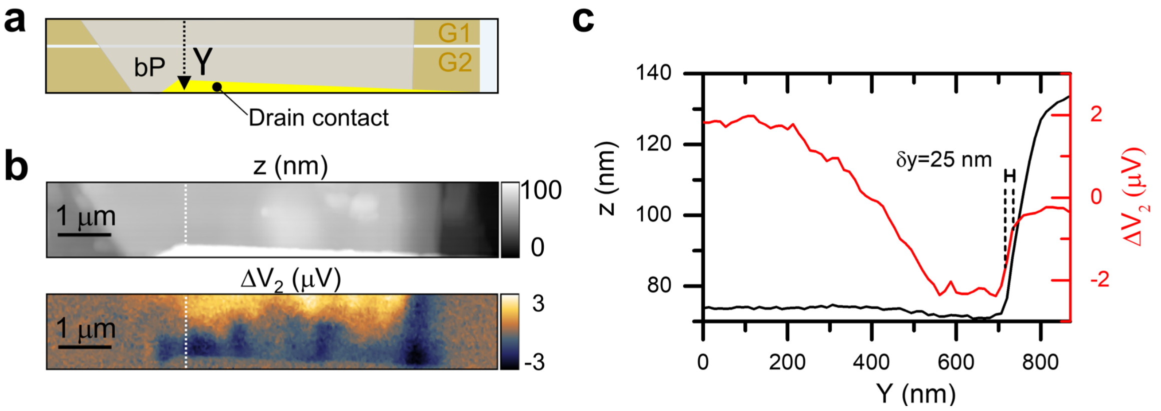

Supplementary figure 12 | Spatial resolution of THz near-field photocurrent nanoscopy. a, Sketch of the sample region on device 1, explored for estimating the near-field photovoltage resolution δy. We analyze line profiles obtained by cutting of the topography and photovoltage maps along the dot arrow line Y, which crosses the interface between the bP heterostructure and the drain contact. **b,** Topography (z), and near-field photovoltage image, ΔV_2, recorded at 2.69 THz (λ = 111 μm). **c,** Topography (black line) and near-field photovoltage ΔV_2 (red line) profiles extracted along the white dashed lines in (b). The spatial resolution δy is estimated using the 20/80 criterion [1] at the drain contact edge located at Y~700 nm, as defined by the topography.

The line profiles of sample topography and photovoltage extracted along the dashed lines are shown in Supplementary Fig. 12b. From the analysis of the line profile of the near-field photovoltage ΔV_2 at the drain contact, we determine a spatial resolution $\delta y = 25$ nm, according to the 20/80 criterion.[1]

The step in sample topography at the drain contact may influence the retrieved estimation of the photovoltage spatial resolution.[7] However, in previous experiments, the photovoltage spatial resolutions estimated at large (~45 nm) and small (~2 nm) topographic jumps were found to be comparable.[1]

Demodulation at higher harmonics of the tapping frequency is commonly used for suppressing the background contribution from the far-field to the near-field signal, exploiting the much stronger dependence on the sample-tip distance of the latter.

In order to choose the demodulation order to analyze, we compare the photovoltage approach curves demodulated at the first, second, and third harmonics of the tapping frequency (see Supplementary Fig.13). Supplementary figure 13 shows the near-field photovoltage approach curves, measured with device 1 excited at 2.69 THz, while keeping the AFM tip in a position where the photovoltage is maximum. The approach curves show a fast signal decay for increasing tip-sample distance, as typical of near-field signals.

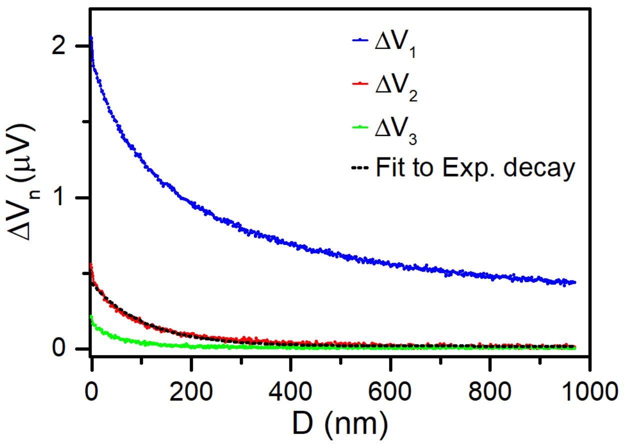

Supplementary figure 13 | Near-field photovoltage approach curves measured on device 1 at 2.69 THz in a position of maximum photovoltage, demodulating at different harmonics order ΔV_n (colored points), together with the fit (black dotted line) of the n= 2 harmonics signal using an exponential decay function y_0+A exp(-D/t), where y_0 is the far-field offset and D the tip-sample distance, from the approach position (D = 0), defined by the zeroing of the mechanical tapping amplitude, to the 80% of the free tapping amplitude. The vertical confinement is estimated from the fitted decay constant t as δ= t/ln(2) and the error is obtained by propagating the error given by the standard deviation of the fit.

The second harmonics is chosen as a good compromise between signal-to-noise ratio and far-field background removal, which is still present in the first harmonics signal. By fitting the decays with an exponential decay function, we obtain an estimation of the THz field vertical confinement at the second harmonics: δ= 77 ± 1 nm. This value is ~ λ_0/1500, with λ_0= 111 μm free-space wavelength of the THz field, indicating a resolution 3 order of magnitude below the diffraction limit.

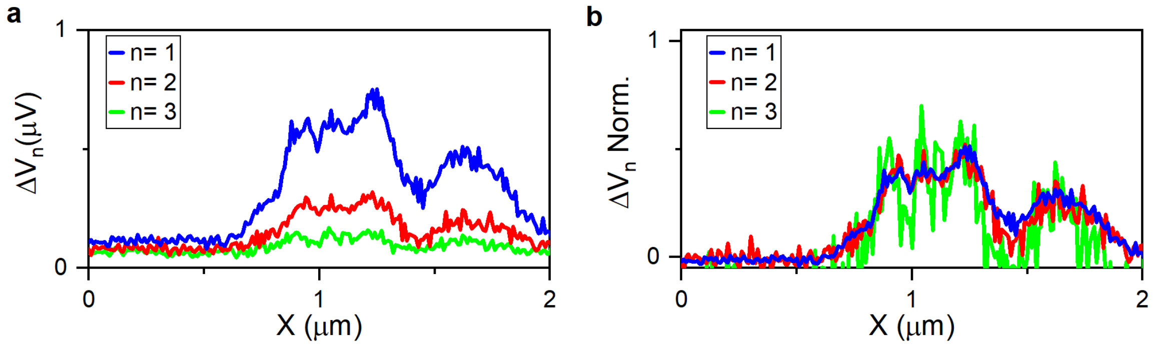

Supplementary figure 14 | Near-field photovoltage at different demodulation orders. a, Near-field photovoltage ΔV_n measured at the junction of the device 3 at different demodulation orders n= 1, 2, 3, using a 2.01 THz QCL, and applying gate voltage V_{G21}= −0.1 V**. b,** Near-field photovoltage profiles in panel (a) after normalization to the maximum, showing same photovoltage periodicity, at the three demodulation orders.

Interestingly, the near-field photovoltage profiles, measured at the different demodulation harmonics, are comparable, showing oscillations with the same periodicity (Supplementary Fig.14). Therefore, a higher demodulation order is not supposed to bring advantages to the device analysis with a clear reduction of the measurement signal-to-noise ratio.

Supplementary Note 5. PTE origin of the photovoltage

To confirm the PTE origin of the observed photovoltage signal, we acquire ΔV_2 maps while varying the carrier densities at the AC junction of device 3 with gates G_{21} and G_{11}, see Supplementary Fig. 15.

We can observe the appearance of sign changes during the gates sweeps at gate voltages far-away from the threshold voltage (see Supplementary Fig.15). The PTE is the only photo-conduction effect that predicts the presence of sign changes even while the sample is not ambipolar, i.e. there is no change from electron to hole conduction.

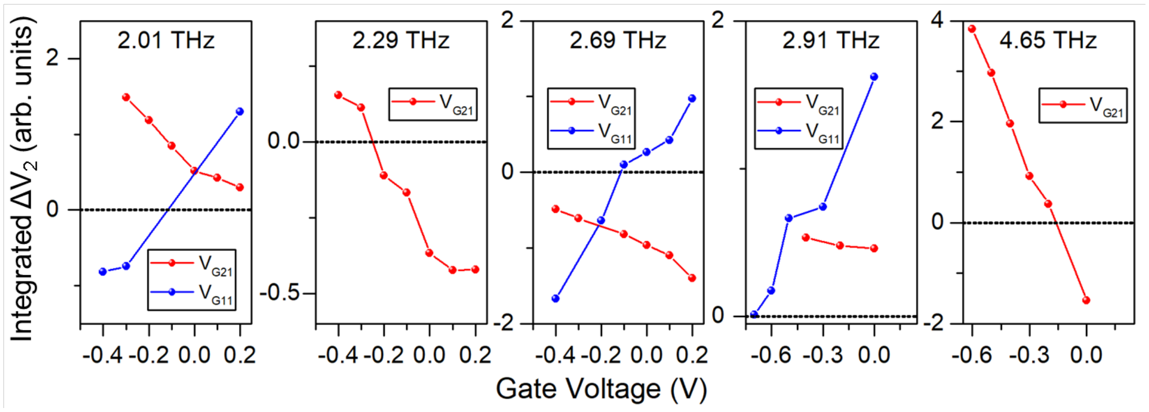

Supplementary figure 15 | Gate dependence of the signal intensity and polarity. Integrated second harmonics near-field photovoltage at the ZZ channel, integrated over a region 1 mm × 10 mm along the AC junction, as a function of V_{G21} (V_{G11}) while keeping G_{11} (G_{21}) grounded, measured at 5 different photon energies.

Specifically, in the explored doping range, we expect contribution to the conduction only from the holes, and the observed sign change can be only explained by a non-monotonic trend of the Seebeck coefficient, which entails a sign change in the quantity $S_b^{21}-S_b^{11}$ that defines the PTE voltage,[9] where S_{11} and S_{21} are the Seebeck coefficients of bP above G_{11} and G_{21}, respectively.

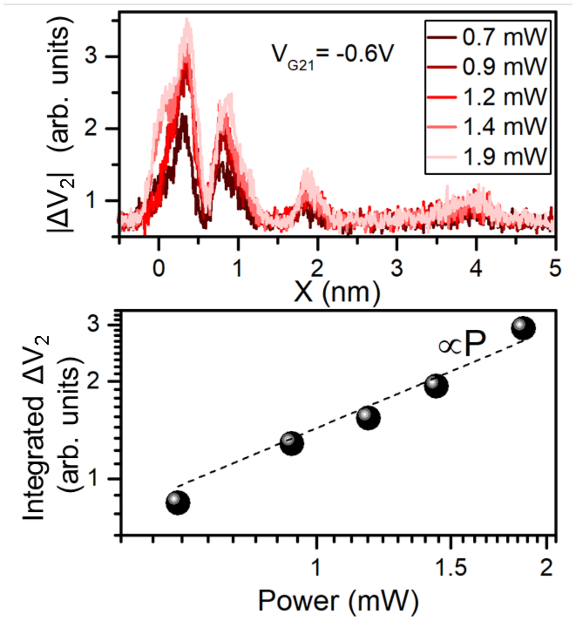

Supplementary figure 16 | Fluence dependence of the PTE current. a, Near-field photovoltage profiles ΔV_2 measured on device 3, illuminated at 4.65 THz, while applying a gate voltage $V_{G21} = -0.6$ V, plotted as a function of the THz-QCL power P. **b,** Integrated signal as a function the THz-QCL power P in log-log plot (dot) as compared with the best linear fitting function (dashed line) f(P)= kP, with k= $(1.44 \pm 0.06)\,mW^{-1}$ and P in mW.

Moreover, we observe an opposite slope while changing V_{G21} or V_{G11}, due to the fact that the difference between the Seebeck coefficients at the junction ($S_b^{21}-S_b^{11}$) assumes opposite sign in the two cases. Finally, we note that the signal variation is more intense under V_{G11} bias, in agreement with the larger transconductance of G_{11} reported in Supplementary Fig.5.

The measured photo-voltage ΔV_2 increases with the incident power, which is varied by tuning the THz-QCL driving current (Supplementary Fig.16). We find a linear dependence, as previously observed by THz photocurrent nanoscopy[10] and as expected for PTE mechanism.

Supplementary Note 6. Modeling of the THz near-field response

6.1 Collective excitation spectrum

The dispersion of PP modes hosted by the bP heterostructure is quantitatively reproduced by the imaginary part of the Fresnel reflection coefficient r_p as a function of frequency ω and in-plane momentum q, which is calculated using the transfer matrix method[11–17] from the different dielectric permittivities ε_i of the six layers composing the sample: 0) the air

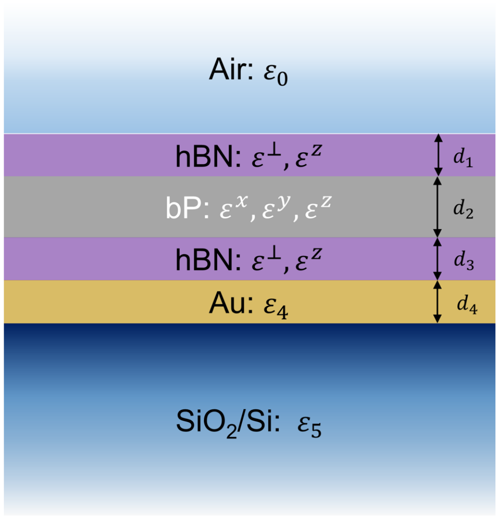

Supplementary figure 17 | Sketch of the multilayer model describing the bP heterostructure. The sample structure consists of an infinite half-space of air, followed by a d_1-thick layer of hBN, a bP layer of thickness d_2, a hBN bottom layer of thickness d_3, a gold thin film of thickness d_4, lying on an infinite half-space of silicon-silica substrate. The dielectric functions ε_i of the layers are also shown in the sketch. In the model we include the uniaxial and biaxial birefringence of hBN and bP.

from which the electromagnetic wave impinges on the sample, 1) the top hBN of thickness d_1; 2) the bP film of thickness d_2; 3) the bottom hBN of thickness d_3; 4) the Cr/Au gate of thickness d_4; 5) the Si/SiO_2 substrate, as sketched in Supplementary Fig. 17. This model includes five interfaces between different dielectric media and the propagation inside four finite-size media. The matrix M is calculated analytically following Ref..[11]:

$$M = \begin{bmatrix} M_{aa} & M_{ba} \\ M_{ab} & M_{bb} \end{bmatrix} = \left(\prod_{i=0}^{3} R_{i,i+1} T_{i+1} \right) R_{4,5}$$

where the matrices $R_{i,i+1}$ describe the reflection at the interfaces between the i and (i+1) layers and are calculated from the Fresnel reflection r_{ij} and transmission t_{ij} coefficients:

$$R_{i,j} = \frac{1}{t_{ij}} \begin{pmatrix} 1 & r_{ij} \\ r_{ij} & 1 \end{pmatrix}, \text{ with } r_{ij} = \frac{\varepsilon_i^{\perp} k_j^z - \varepsilon_j^{\perp} k_i^z}{\varepsilon_i^{\perp} k_j^z + \varepsilon_j^{\perp} k_i^z}$$

Where $\varepsilon_i^{\perp}$ are the in-plane dielectric permittivity and k_i^z is the out-of-plane *k*-vector of the electromagnetic wave in layer i. The latter is related to the in-plane polariton momentum *q* and to the in-plane and out-of-plane permittivities $\varepsilon_i^{\perp}$ and ε_i^z as[17]:

$$k_i^z = \sqrt{\varepsilon_i^{\perp} \left(\frac{\omega^2}{c^2} - \frac{q^2}{\varepsilon_i^z} \right)}$$

The matrices T_i describe the phase delay accumulated in the propagation through the layer i, which depends on the layer thickness d_i, as follows:

$$T_{i,j} = \begin{pmatrix} exp\,(ik_i^z d_i) & 0 \\ 0 & exp\,(-ik_i^z d_i,) \end{pmatrix}$$

Finally, the reflection coefficient is obtained from the ratio $r_p = M_{ba}/M_{aa}$ of the transfer matrix (M) elements as follows:

$$M_{aa} = r_{45} e^{-ik_4 d_4} \{ e^{-ik_3 d_3} [r_{23} e^{ik_2 d_2} (r_{01} e^{-ik_1 d_1} r_{12} + e^{ik_1 d_1}) + e^{-ik_2 d_2} (r_{01} e^{-ik_1 d_1} + r_{12} e^{ik_1 d_1})] + r_{34} e^{ik_3 d_3} [e^{-ik_2 d_2} r_{23} (r_{01} e^{-ik_1 d_1} + r_{12} e^{ik_1 d_1}) + e^{ik_2 d_2} (r_{01} e^{-ik_1 d_1} r_{12} + e^{ik_1 d_1})] \} + e^{ik_4 d_4} \{ r_{34} e^{-ik_3 d_3} [r_{23} e^{ik_2 d_2} (r_{01} e^{-ik_1 d_1} r_{12} + e^{ik_1 d_1}) + e^{-ik_2 d_2} (r_{01} e^{-ik_1 d_1} + r_{12} e^{ik_1 d_1})] + e^{ik_3 d_3} [e^{-ik_2 d_2} r_{23} (r_{01} e^{-ik_1 d_1} + r_{12} e^{ik_1 d_1}) + e^{ik_2 d_2} (r_{01} e^{-ik_1 d_1} r_{12} + e^{ik_1 d_1})] \}$$

$$M_{ba} = r_{45}e^{-ik_4d_4}\{e^{-ik_3d_3}[r_{23}e^{ik_2d_2}(r_{01}e^{ik_1d_1} + r_{12}e^{-ik_1d_1}) + e^{-ik_2d_2}(r_{01}r_{12}e^{ik_1d_1} + e^{-ik_1d_1})] + r_{34}e^{ik_3d_3}[e^{-ik_2d_2}r_{23}(r_{12}r_{01}e^{ik_1d_1}+e^{-ik_1d_1}) + e^{ik_2d_2}(r_{01}e^{ik_1d_1} + r_{12}e^{-ik_1d_1})]\} + e^{ik_4d_4}\{r_{34}e^{-ik_3d_3}[r_{23}e^{ik_2d_2}(r_{01}e^{ik_1d_1} + r_{12}e^{-ik_1d_1}) + e^{-ik_2d_2}(r_{01}r_{12}e^{ik_1d_1} + e^{-ik_1d_1})] + e^{ik_3d_3}[e^{-ik_2d_2}r_{23}(r_{01}r_{12}e^{ik_1d_1} + e^{-ik_1d_1}) + e^{ik_2d_2}(r_{01}e^{ik_1d_1} + r_{12}e^{-ik_1d_1})]\}$$

To evaluate the out-of-plane momentum *k* and the reflection coefficients, we use the layer thicknesses d_i determined by AFM imaging of the sample, and we calculate the dielectric permittivities, as detailed below.

The theoretical r_p map for device 3 (i.e. for the hBN (10 nm)/ bP (15 nm)/ hBN (19 nm) heterostructure) is reported in Supplementary Fig. 18, as compared with the experimental dispersion.

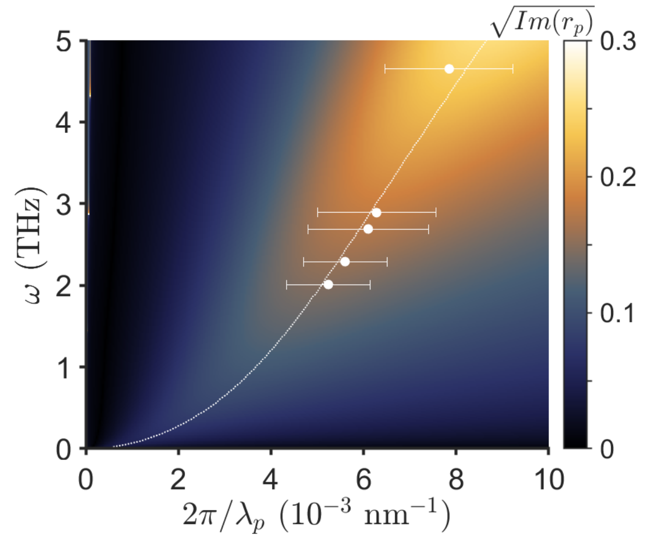

Supplementary figure S18 | Dispersion of THz plasmon polariton in the device 3. Theoretical calculation of the imaginary part of the Fresnel reflection coefficient r_p for p-polarized light (colored map) compared with the experimental dispersion measured by THz photocurrent nanoscopy (white dots). The theoretical map is evaluated with {n, μ} = {1 ×10^{19} cm^{-3}, 130 cm^2/Vs}. Error bars are evaluated as the standard deviation extracted from the fit of the photovoltage line profiles.

6.2 Dielectric permittivities of the bP based heterostructure

The propagation in air is described by the isotropic dielectric permittivity $\varepsilon_0$which, in CGS units, is assumed as equal to 1. The SiO_2/Si substrate (layer 5) is described by an isotropic average permittivity ε_5= 6.3, while for the gold film describing the gates we use ε_5= −2

$\times 10^5 - 4\times 10^6$i. The light propagation in the bottom and top hBN is described taking into account the uniaxial anisotropy ($\varepsilon^{\perp} \neq \varepsilon^{z}$) of hBN given by its mid-infrared hyperbolic phonons. The Reststrahlen bands of hBN are at energies (RS1 = 780 to 830 cm^{-1}; RS2 = 1370 to 1610 cm^{-1}) far above the investigated range, such that we can assume constant in-plane and out-of-plane values[3]: $\varepsilon_1^{\perp} = \varepsilon_3^{\perp} = 8.5$ and $\varepsilon_1^{z} = \varepsilon_3^{z} = 3.5$.

The dielectric permittivity of bP has biaxial anisotropy $\varepsilon_x \neq \varepsilon_y \neq \varepsilon_z$ given by the puckered structure of bP. The dielectric permittivities in the different directions l = x, y, z corresponding to zigzag (x), armchair (y) and stacking (z) directions, are evaluated using the Drude model:

$$\varepsilon_l = \frac{-\omega_D^2}{(\omega^2 + i\omega\gamma_l)}$$

$\omega_D^l = \sqrt{\frac{ne^2}{\varepsilon_0 m_l^*}}$ is the plasma frequency, which depends on the carrier density n and on the effective mass of holes (bP is p-doped) m_l^*, which strongly varies along the different crystallographic directions: $m_{AC}^* = 0.36\ m_0$, $m_{ZZ}^* = 0.81\ m_0$, $m_z^* = 0.09\ m_0$.

The effective masses and the bandgap of bP are expected to show strong thickness dependence [18] for atomic layer number N < 10 corresponding to thicknesses of < 6 nm. Since we are dealing with much thicker flakes (15-29 nm), we neglect the thickness dependence and assume the same values for the anisotropic effective mass tensor of the three devices.

The scattering rate $\gamma_l = e/\mu m_l^*$ is evaluated using its relation with the effective mass m_l^* and the mobility μ.

The in-plane permittivity in the bP layer $\varepsilon_2^{\perp}$ is evaluated from the $\varepsilon_{l=AC}$ and $\varepsilon_{l=ZZ}$ considering the angle θ of the polariton propagation with respect to the armchair direction:

$$\varepsilon_2^{\perp} = \varepsilon_2^{AC} cos^2\theta + \varepsilon_2^{ZZ} sin^2\theta$$

The carrier density n and the mobility μ of the three reported samples are considered as fitting parameters, optimized by minimizing the difference between the calculated polariton dispersion and the experimental dispersion points.

The permittivities that reproduce the experimental PP dispersion are reported in Supplementary Fig. 19.

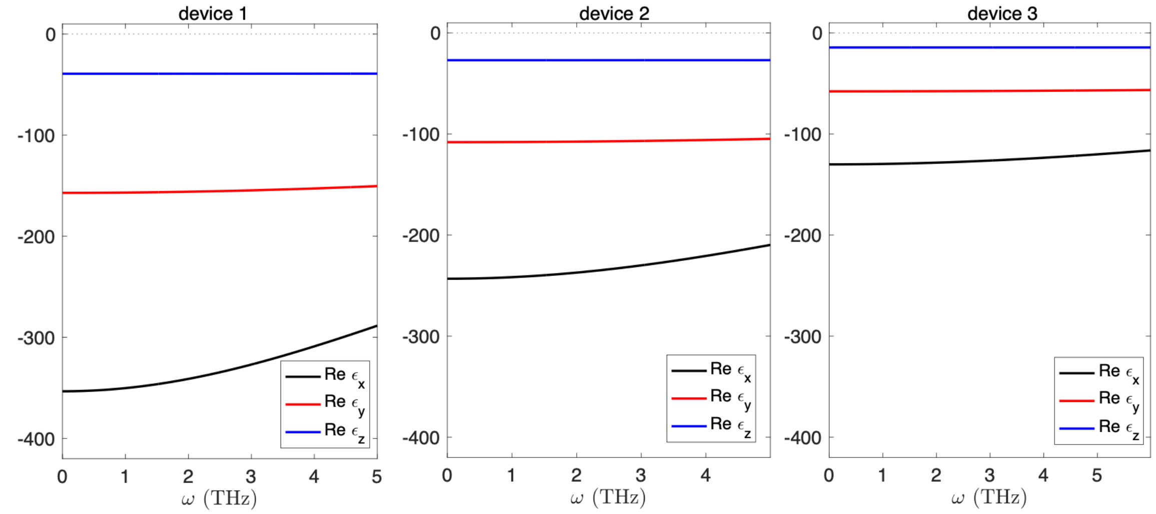

Supplementary figure 19 | Calculated anisotropic dielectric permittivities of bP in the heterostructures 1-3.

6.3 Polariton dispersion dependence on bP carrier density and mobility

The carrier density and mobility have a major impact on the dispersion of plasmon polaritons, hence the orientation of the bP crystals cannot be solely inferred from the photocurrent measurements.

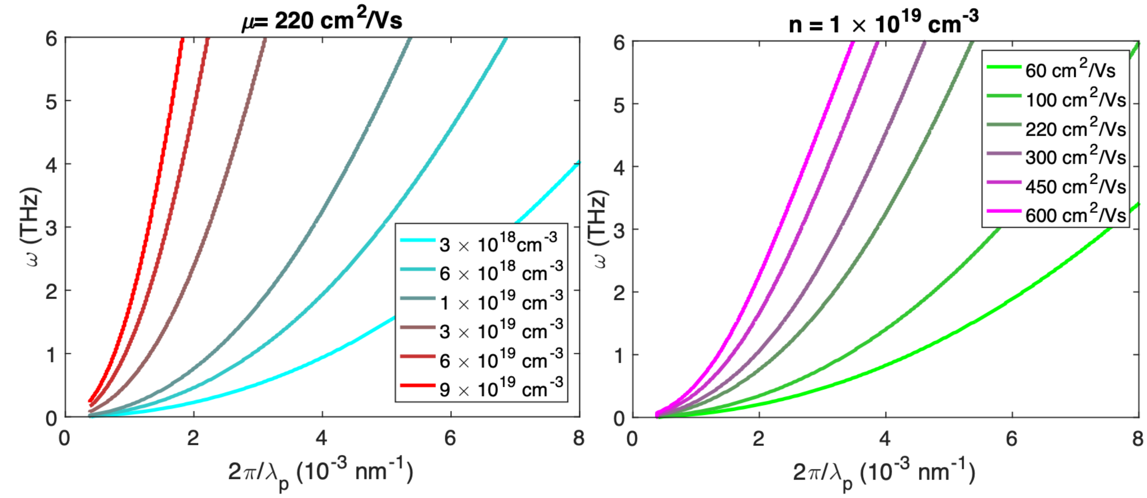

Supplementary figure S20 | Simulated dispersion for device 1 obtained varying n while keeping fixed carrier mobility, μ= 220 cm^2/Vs (a), and for different μ keeping fixed carrier density n = 1 $\times 10^{19}$ cm^{-3} (b).

Supplementary Fig.20 shows the simulated dispersion for device 1, obtained using different values of carrier density n and mobility μ, retrieved from the literature. The impact on the effective dispersion curve is comparable and even larger than that of the crystal orientation, which here was estimated using Raman measurements.

Supplementary Note 7. Device optimization for photocurrent measurements

The AFM tips, optimized to efficiently scatter THz radiation, are generally quite long. The nominal length of the tips we used is 80 µm. It is important to consider possible obstacles in approaching such long tips to the sample. Photocurrent nanoscopy measurements require the presence of electrical readouts. A two-gates transistor configuration has at least four contacts on the sample (source-drain and the gates), and four-gates devices have at least seven contacts (two sources, one drain, four gates). Therefore, it is very important to design the contacts such that the bonding wires do not prevent the tip approaching the FET channel, avoiding any accidental contact with the tip while searching for the region of interest. In Supplementary Fig.21, we report a SEM image showing an example of a two-terminal device with the large gold bonding pads, all placed on one side, such that the tip can be approached from the opposite side.

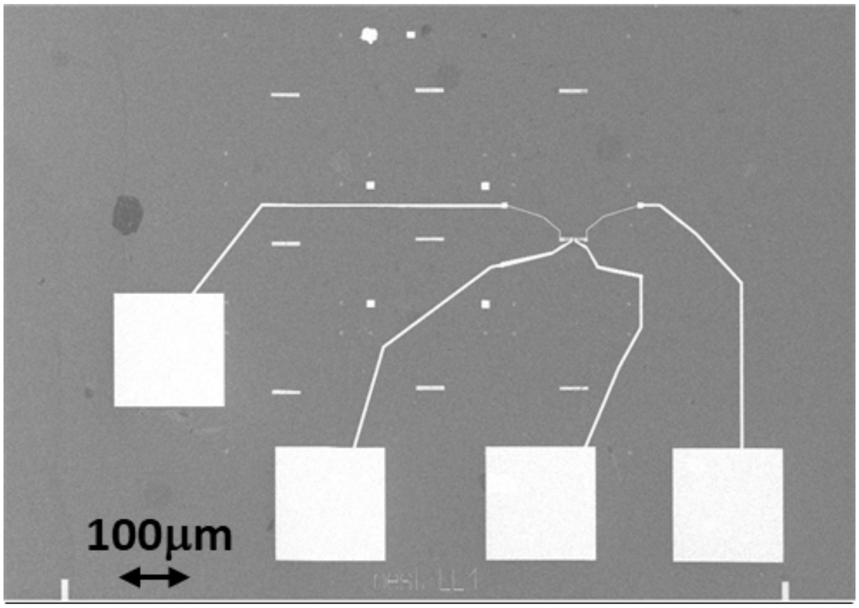

Supplementary figure S21 | SEM image of a two-terminal device comprising 4 electrical contacts designed to allow easy access to the AFM tip from the top.

7.1 Tip electrostatic charging

To tune the charge carrier density of the device, we apply gate voltages in the range of hundreds mV to few Volts. Given the small sample-to-tip distance, the AFM metal tip can accumulate charge via electrostatic induction. The tip charging can severely affect the imaging quality producing distortions, because it modifies the restoring force acting on the tip. Moreover, if the voltage drop between the tip and the gap is too large and exceeds the breakdown voltage of the system hBN/bP/hBN/air, the hBN bottom layer can break and eventually explode due to the excessive heat produced by intense localized current flow. The breakdown is more likely to occur at large gate voltages (> 2 V), large tapping amplitudes and thinner hBN bottom layers (< 20 nm). An example of dielectric explosion is reported in Supplementary Fig. 22, occurred while the AFM tip was not grounded, operated at tapping amplitudes > 500 nm, scanning an heterostructure with bottom hBN thickness of 10 nm, while applying a gate voltage >2V. Operating the tip at large tapping amplitudes (> 200 nm) is detrimental due to the modulation of the capacitive coupling between the tip and the gates. To minimize the risk of breakdown, we suggest to first approach the tip to the sample with the device grounded, and then gradually increase the gate voltages.

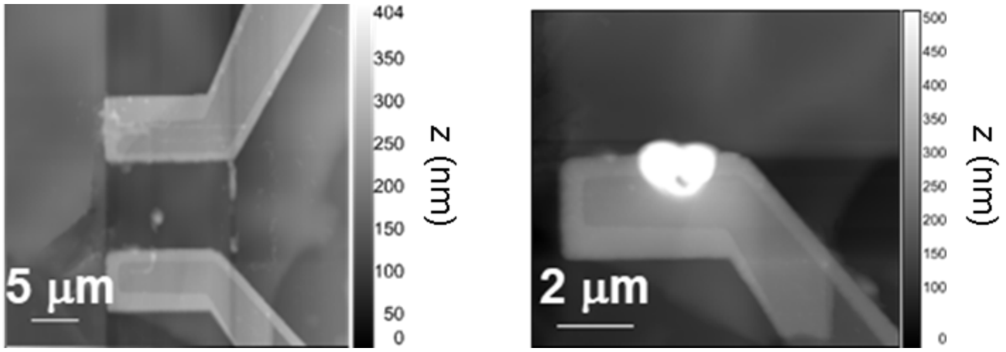

Supplementary figure S22 | AFM images of a two-gate FET before (left) and after (right) the explosion of the dielectric. Topography in nm.

It is crucial to maintain all contacts at the same ground, which should also coincide with the ground of the SNOM acquisition card and tip. To ground the tip, we use silver paste to electrically connect the tip cantilever to the tip holder. To reduce electrostatic and acoustic noise, the purging box was covered, internally, by sound absorbing foam, and externally,

by a 3 mm thick aluminum case with a 2 inches' circular aperture for the THz beam, covered by 1 mm TPX window. The window was slightly tilted to avoid the reinjection of the reflected beam into the THz-QCL.

Previous measurements of photoconduction mechanism in bP has revealed a finite contribution from bolometric effect[19,20], which is related to the light induced modulation of the lattice temperature. If the conductivity depends on the lattice temperature, the tip-induced lattice temperature local variation can be sensed as a variation in the source-drain current. It would be interesting to observe the plasmon polariton interferometric pattern as a modulation of the bolometric photocurrent. In order to see the bolometric current, it is crucial to apply a finite bias to the channel. The applied bias is an additional control knob to exploit to increase the technique sensitivity. We concentrate on PTE photovoltage since it requires any bias and therefore minimizes the device power consumption.

Supplementary Note 8. Raman spectra

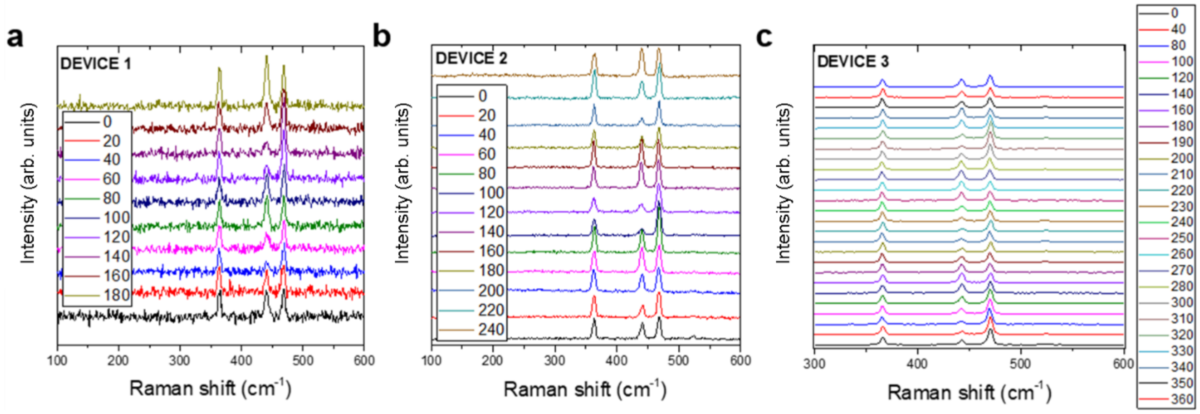

Supplementary figure S23 | Raman spectra measured in the three devices with excitation wavelength 638 nm at different angle with respect to the junction line. (a) device 1, (b) device 2, (c) device 3.

In Supplementary Fig.24 we show the polar plot used to determine the crystallographic orientation of the bP flake included in device 2, as shown in Figure 2b and here plot on a 360° range.

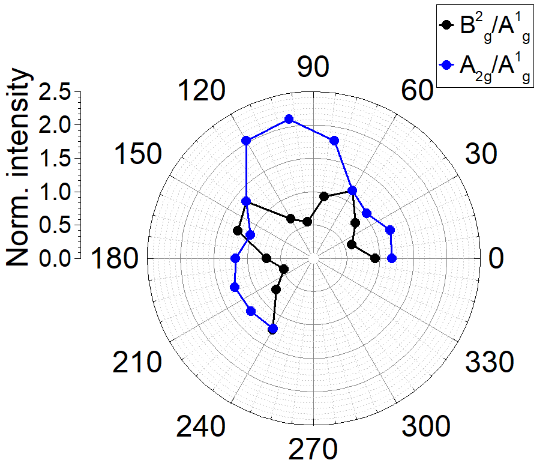

Supplementary figure 24 | Angle-resolved Raman spectroscopy. Raman peak intensity of the mode B_{2g} and A^2_g normalized to the intensity of the A^1_g mode as a function of the angle in between the incident pump polarization and the junction line of device 2 (hBN (6 nm)/bP (29 nm)/hBN (16 nm)).

Supplementary Note 9. Measurement of the bP polaritons wavelength

9.1 Analysis of the near-field photovoltage profiles of Figure 2

The momentum k_p and the wavelength λ_p of the THz polaritons of bP are extracted from the periodicity of the near-field photovoltage ΔV_2 line profiles perpendicular to the p-n junctions. The underneath assumption is that the polariton waves are launched by the s-SNOM tip and reflected at the flake edges, so that the period of the ΔV_2 oscillations corresponds to half of the polariton wavelength, $\lambda_p/2$. As previously reported in graphene,[1] the PTE near-field signal of bP contains a background signal arising either from the non-plasmonic heating of the charge carriers or from local plasmonic heating. In both cases such a signal does not contribute to the oscillating component of the signal due to the propagating plasmon polaritons. To isolate the latter contribution, we subtract the signal background before fitting the photovoltage profiles with a damped sinusoidal function. To this aim, we evaluate the Fourier transform (FT) of the line profiles of Figure 2, using zero padding and triangular apodization. The moduli of the FT of the line profiles of Figure 2c, 2d are reported in Supplementary Fig.25a and Supplementary Fig.26a, respectively. The background is subtracted, setting a high-pass filter, corresponding to the shaded areas in Supplementary Fig.25 and 26. The peaks attributed to the propagating polaritons are marked

with red dotted lines for an easier comparison with the dispersion curves shown in Figure 2.

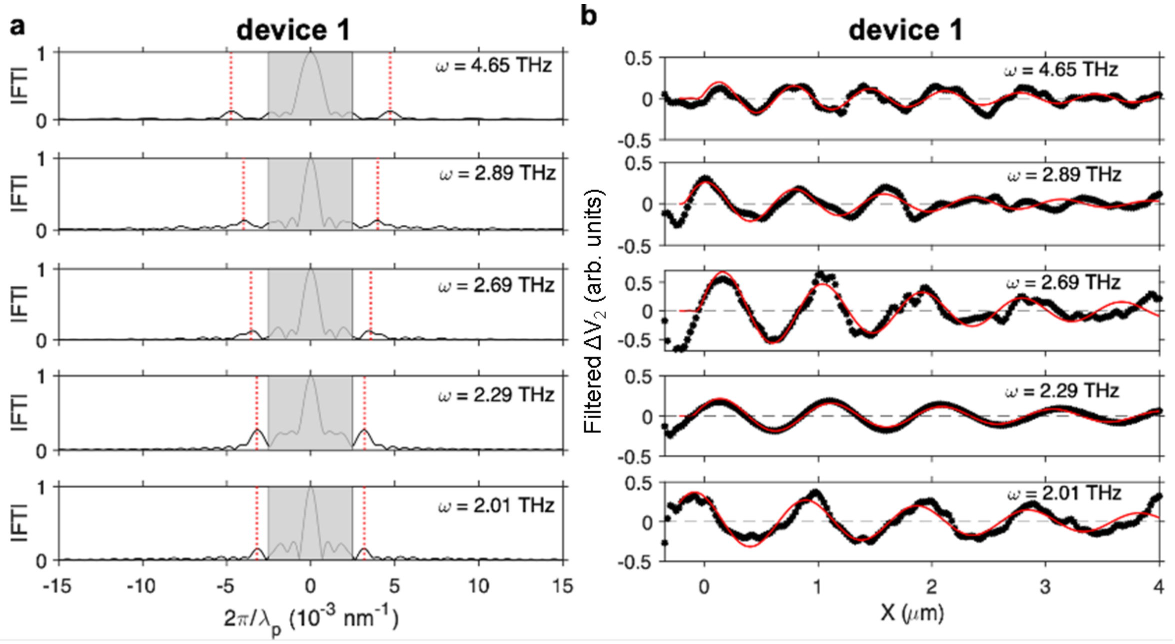

Supplementary figure 25 | Fitting of the near-field photovoltage line profiles of device 1. a) Moduli (black solid lines) of the Fourier transform (FT) of the near-field photovoltage profiles of device 1, reported in Figure 2c, measured at different frequencies from 2.01THz (bottom panel) to 4.65 THz (upper panel). The FTs are shown as a function of the polariton momentum k_p (the spatial frequency multiplied by two). The curves are normalized to the maximum. Grey shaded areas indicate the frequency ranges filtered out to subtract the background due to local heating. Red dotted lines identify the position of the peak attributed to the propagating polaritons. b) Near-field photovoltage profiles after the background subtraction (black dots), as a function of the spatial coordinate, together with the damped sinusoidal fitting function (red solid line) used to extract the polariton momentum and the dispersion curve of device 1 in Figure 2e.

Supplementary Fig.25b and Supplementary Fig.26b plot the near-field photovoltage profiles after background subtraction together with the fit of the real-space line profiles used to extract the oscillation periodicity.

It is worth mentioning that the attenuation of the PTE signal can be attributed to several factors, including the temperature profile, that depends on the plasmon induced heating, and the Seebeck coefficient that depends on the carrier density profile induced by electrostatic gating.

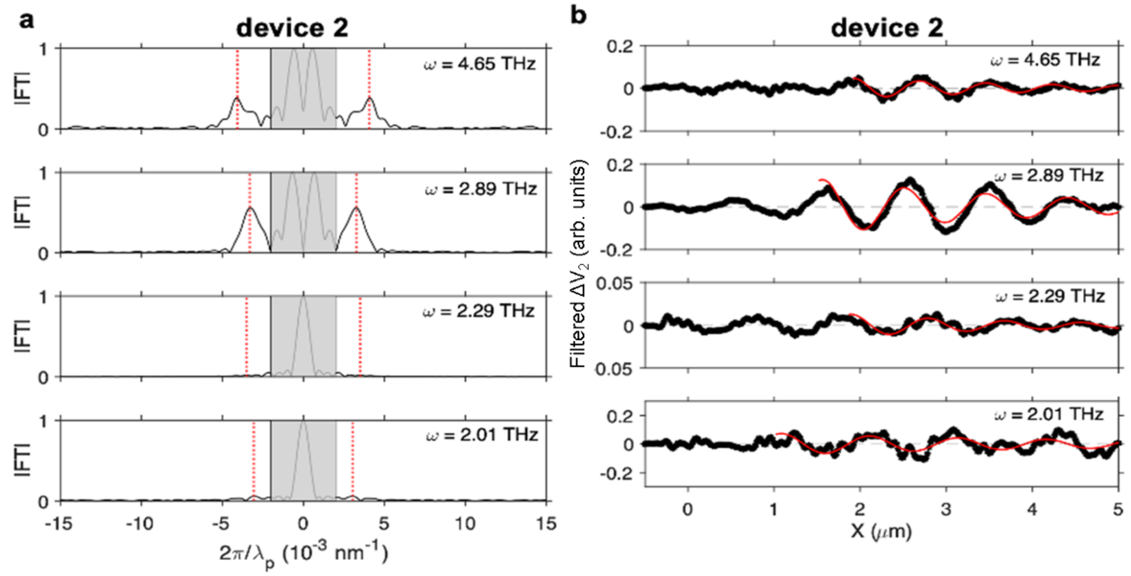

Supplementary figure 26 | Fitting of the near-field photovoltage line profiles of device 2. a, Moduli (black solid lines) of the Fourier transform (FT) of the near-field photovoltage profiles of device 2, reported in Figure 2d, measured at different frequencies from 2.01 THz (bottom panel) to 4.65 THz (upper panel). The FT are shown as a function of the polariton momentum k_p (the spatial frequency multiplied by two). The curves are normalized to the maximum. Grey shaded areas indicate the frequency ranges filtered out to subtract the background due to local heating. Red dotted lines identify the position of the peak attributed to the propagating polaritons. **b,** Near-field photovoltage profiles after the background subtraction (black dots), as a function of the spatial coordinate, together with the damped sinusoidal fitting function (red solid line) used to extract the polariton momentum and the dispersion of device 1 in Figure 2f.

9.2 Analysis of the near-field photovoltage profiles of Figure 4

Supplementary Fig. S27, shows the near-field photovoltage ΔV_2 profiles measured in device 3 along the AC direction, while applying a finite gate voltage V_{G11}=-0.4, -0.2 V, together with the damped sinusoidal fitting functions, adopted to extract the dispersion characteristic of Figure 4c, main text.

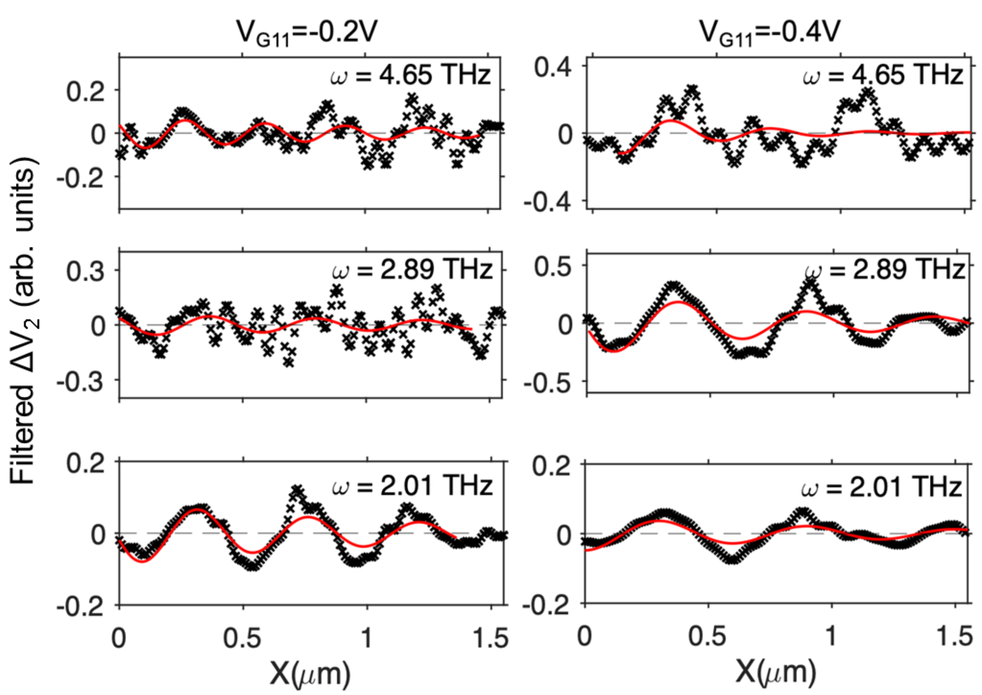

Supplementary figure 27 | Fitting of the near-field photovoltage line profiles of device 3. Spatial profile of the near-field photovoltage after the background subtraction (black dots), plotted together with the damped sinusoidal fitting function (red solid line) used to extract the polariton momentum and the dispersion characteristic of device 1, reported in Figure 4c (main text), while applying a finite gate voltage V_{G11}=-0.2 V (left panels), and V_{G11}=-0.4V (right panels).

Supplementary Note 10. Numerical simulations of s-SNOM polariton interferometric patterns

We perform a full-wave simulation (Comsol Multiphysics) of the electric field distribution in the investigated bP devices, including the actual flake size and electrodes distances. The s-SNOM tip illuminated by the THz QCLs is modelled as a vertically oriented dipole source placed above the device surface at a distance z = 150 nm, as conventionally done for predicting the near-field of an elongated AFM tips illuminated by p-polarized light [21-23].

We calculate the component of the electric field perpendicular to the sample E_z(x,y), at a distance z= 50 nm from the surface, as a function of the dipole position in the xy plane, see Supplementary Fig.28.

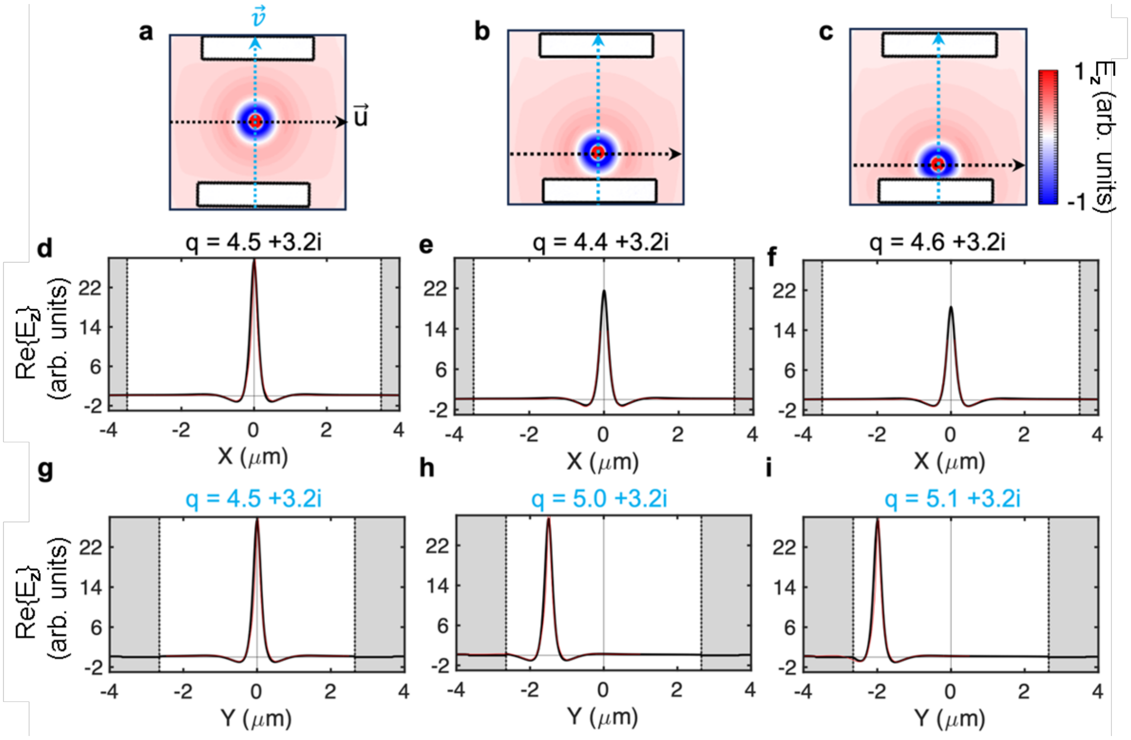

Supplementary figure 28 | Simulation of the near-field distribution of polaritons in device 1at 2 THz frequency. a-c, *Real part of the out-of-plane component of the electric field Re{E_z} at z=50 nm, as generated by a vertically oriented electric dipole placed at z =150 nm in different x-y positions: at the center of the transistor channel (a), at 1.2 μm from the bottom electrode (b), at 600 nm from the bottom electrode.* ***d-f,*** *Electric field (black solid line) along the black dashed line* ***u*** *perpendicular to the junction (parallel to the electrodes) extracted from the map in panel a (d), panel b (e) and panel c (f), together with the fit (red solid line) with the function Re{A exp(i q*X)/√X}, where q is the complex-valued polariton momentum such that k_p= Re{q}.* ***g-i,*** *Electric field (black solid line) along the blue dashed line* ***v*** *parallel to the junction (perpendicular to the electrodes) extracted from the map in panel a (g), panel b (h) and panel c (i), together with the fit (red solid line) with the function Re{A exp(i q*Y)/√Y}, where q is the complex-valued polariton momentum. The* ***u*** *direction forms an angle of -17° with the zigzag direction of bP which is taken along the x axis.*

The multilayer structure of our devices is taken into account including 8 different material domains: the silicon substrate (thickness 100 μm, isotropic relative permittivity 6.3), a gold layer describing the back gates (thickness 30 nm, refractive index *n*=1380+i1450), the bottom and top hBN layers (thickness 19 and 10 nm, refractive index 2.2+0.005i (in-plane direction) and 2.05+0.005i (out of plane direction)), the bP flake, two gold source-drain electrodes (thickness 45 nm, same refractive index as the gate), the air surrounding the device, isotropic relative permittivity 1). The bP flake is described by a diagonalized dielectric tensor using the complex-value dielectric functions along the zigzag (x) armchair (y) and stacking (z) directions, used for the simulation of the reflectivity of device 1, as shown in Supplementary Fig. 20. To model device 1, we introduce a bP flake of thickness

d = 10 nm, rectangular lateral size 7 μm × 13μm, rotated by 17° with respect to the zigzag axis. We placed the electrodes at a relative distance of 6.3 μm to evaluate the impact of the interferometric pattern generated by propagating polaritons that we measure at the p-n junction by PTE detection.

Supplementary References

1. Alonso-González, P. *et al*. Acoustic terahertz graphene plasmons revealed by photocurrent nanoscopy. *Nat. Nanotechnol.* **12**, 31–35 (2016).

2. Lee, M. *et al*. Ultrahigh Anisotropic Transport Properties of Black Phosphorus Field Effect Transistors Realized by Edge Contact Finally, a scattering phase diagram is demonstrated to understand the role of different scattering mechanisms on the modulation of mobility anisotropy in BP FETs with edge and top contacts. (2021). doi:10.1002/aelm.202100988

3. Mishchenko, A. *et al*. Nonlocal Response and Anamorphosis: The Case of Few-Layer Black Phosphorus. *Nano Lett.* **15**, 6991–6995 (2015).

4. Laturia, A., Van De Put, M. L. & Vandenberghe, W. G. Dielectric properties of hexagonal boron nitride and transition metal dichalcogenides: from monolayer to bulk. *npj 2D Mater. Appl.* **2**, 6 (2018).

5. Mitrofanov, O. *et al*. Near-field terahertz probes with room-temperature nanodetectors for subwavelength resolution imaging. *Sci. Rep.* **7**, 44240 (2017).

6. Giordano, M. C. *et al*. Phase-resolved terahertz self-detection near-field microscopy. *Opt. Express* **26**, 18423–18435 (2018).

7. Pogna, E. A. A. *et al*. Terahertz near-field nanoscopy based on detectorless laser feedback interferometry under different feedback regimes. *APL Photonics* **6**, 61302 (2021).

8. Gamage, S. *et al*. Nanoscopy of Black Phosphorus Degradation. *Adv. Mater. Interfaces* 1600121 (2016).

9. Viti, L., Politano, A., Zhang, K. & Vitiello, M. S. Thermoelectric terahertz photodetectors based on selenium-doped black phosphorus flakes. *Nanoscale* **11**, 1995–2002 (2019).

10. Pogna, E. A. A. A. *et al*. Unveiling the detection dynamics of semiconductor nanowire photodetectors by terahertz near-field nanoscopy. *Light Sci. Appl.* **9**, 1–12 (2020).

11. Huber, M. A. *et al*. Femtosecond photo-switching of interface polaritons in black phosphorus heterostructures. *Nat. Nanotechnol.* **12**, 207–211 (2017).

12. Fei, Z. *et al*. Infrared Nanoscopy of Dirac Plasmons at the Graphene–SiO2 Interface. *Nano Lett*. **11**, 4701–4705 (2011).

13. Dai, S. *et al*. Tunable phonon polaritons in atomically thin van der Waals crystals of boron nitride. *Science* **343**, 1125–1129 (2014).

14. Caldwell, J. D. *et al*. Atomic-scale photonic hybrids for mid-infrared and terahertz nanophotonics. *Nature Nanotechnology* **11**, 9–15 (2016).

15. Woessner, A. *et al*. Highly confined low-loss plasmons in graphene-boron nitride heterostructures. *Nat. Mater*. **14**, 421–425 (2015).

16. Yoxall, E. *et al*. Direct observation of ultraslow hyperbolic polariton propagation with negative phase velocity. *Nat. Photonics* **9**, 674–678 (2015).

17. Wu, J. S., Basov, D. N. & Fogler, M. M. Topological insulators are tunable waveguides for hyperbolic polaritons. *Phys. Rev. B - Condens. Matter Mater. Phys.* **92**, 1–12 (2015).

18. Chaves, A., Ji, W., Maassen, J., Dumitrică, T. & Low, T. Theoretical Overview of Black Phosphorus. in *2D Materials* 381–412 (Cambridge University Press, 2017). doi:10.1017/9781316681619.022

19. Low, T., Engel, M., Steiner, M. & Avouris, P. Origin of photoresponse in black phosphorus phototransistors. *Phys. Rev. B* **90**, 81408 (2014).

20. Viti, L. *et al*. Efficient Terahertz detection in black-phosphorus nano-transistors with selective and controllable plasma-wave, bolometric and thermoelectric response. *Sci. Rep*. **6**, 20474 (2016).

21. Chen, S., Bylinkin, A., Wang, Z. et al. Real-space nanoimaging of THz polaritons in the topological insulator Bi_2Se_3. *Nat Commun* 13, 1374 (2022).

22. Nikitin, A. Y. et al. Real-space mapping of tailored sheet and edge plasmons in graphene nanoresonators. *Nat. Photon*. **10**, 239–243 (2016).

23. Ma, W. et al. In-plane anisotropic and ultra-low-loss polaritons in a natural van der Waals crystal. *Nature* **562**, 557–562 (2018).

24. H.-J. Hagemann, W. Gudat, and C. Kunz. Optical constants from the far infrared to the x-ray region: Mg, Al, Cu, Ag, Au, Bi, C, and Al_2O_3. *J. Opt. Soc. Am*. **65**, 742-744 (1975
